# Recent human evolution has shaped geographical differences in susceptibility to disease

**DOI:** 10.1186/1471-2164-12-55

**Published:** 2011-01-24

**Authors:** Urko M Marigorta, Oscar Lao, Ferran Casals, Francesc Calafell, Carlos Morcillo-Suárez, Rui Faria, Elena Bosch, François Serra, Jaume Bertranpetit, Hernán Dopazo, Arcadi Navarro

**Affiliations:** 1 Institute of Evolutionary Biology (UPF-CSIC), PRBB, Doctor Aiguader 88, 08003, Barcelona, Catalonia, Spain; 2 Department of Forensic Molecular Biology, Erasmus University Medical Center Rotterdam, 3000 CA Rotterdam, The Netherlands; 3 CIBER en Epidemiologia y Salud Pública (CIBERESP), Barcelona, Catalonia, Spain; 4 National Institute for Bioinformatics, Universitat Pompeu Fabra, Barcelona, Spain; 5 CIBIO, Centro de Investigação em Biodiversidade e Recursos Genéticos, Universidade de Porto, Campus Agrário de Vairão, 4485 - 661, Vairão, Portugal; 6 Pharmacogenomics and Comparative Genomics Unit, Centro de Investigación Príncipe Felipe (CIPF), Valencia 46012, Spain; 7 Institució Catalana de Recerca i Estudis Avançats (ICREA), Barcelona, Catalonia, Spain

## Abstract

**Background:**

Searching for associations between genetic variants and complex diseases has been a very active area of research for over two decades. More than 51,000 potential associations have been studied and published, a figure that keeps increasing, especially with the recent explosion of array-based Genome-Wide Association Studies. Even if the number of true associations described so far is high, many of the putative risk variants detected so far have failed to be consistently replicated and are widely considered false positives. Here, we focus on the world-wide patterns of replicability of published association studies.

**Results:**

We report three main findings. First, contrary to previous results, genes associated to complex diseases present lower degrees of genetic differentiation among human populations than average genome-wide levels. Second, also contrary to previous results, the differences in replicability of disease associated-loci between Europeans and East Asians are highly correlated with genetic differentiation between these populations. Finally, highly replicated genes present increased levels of high-frequency derived alleles in European and Asian populations when compared to African populations.

**Conclusions:**

Our findings highlight the heterogeneous nature of the genetic etiology of complex disease, confirm the importance of the recent evolutionary history of our species in current patterns of disease susceptibility and could cast doubts on the status as false positives of some associations that have failed to replicate across populations.

## Background

The discovery of genetic variants that increase susceptibility to disease represents one of the greatest challenges for epidemiology and genomics [1]. Detailed knowledge about the etiology of many diseases keeps accumulating and in the near future it will help to improve disease management [2]. After decades of research in genetic epidemiology, more than 51,000 different association studies for human diseases have been published and 11,501 genes have been described to be associated to disease, as recorded up to December 2010 in the HuGENet browser [3]. Moreover, thanks to last technological advances, we have recently escalated into a flurry of genome-wide association studies (GWAS) that simultaneously study hundreds of thousands of SNPs over the whole genome [3-5]. For instance, most GWAS recorded in the HuGENet browser have been published recently, from 2008 on (812 out of 935 by December 15^th^, 2010).

In spite of their success, genetic association studies for common complex diseases usually suffer from a problem of lack of reproducibility of results. Only a very low number of risk variants have been shown to present a consistent pattern of positive replication through independent studies [4-8]. Different confounding factors may constitute the source of these inconsistencies. Two well-known sources of lack of replicability are reduced statistical power due to small (and varying) experimental samples sizes [5,9,10]; and population stratification [11]. Other potential sources of lack of replicability include disease heterogeneity, since some complex diseases might include similar entities with shared symptoms but different genetic architectures [12]; hidden age-varying effects [13]; biased ascertainment of genetic markers [14] and publication bias [7]. To overcome these confounding factors, the NCI-NHGRI working group on replication in association studies published a set of recommendations to achieve essential credibility of true positive disease-associated genetic variants [4]. One of their crucial recommendations is that the replication of results in an independent sample of individuals is required to make an association statistically trustable.

However, a true association could fail to be replicated due to heterogeneity in the genetic architecture of the disease under study, particularly when replicas are carried out in populations with different evolutionary histories. Indeed, many common SNPs present significantly different frequencies among human populations or even appear to be polymorphic just in certain populations (i.e. they are population-specific SNPs) [15]. For instance, the six possible pairwise comparisons of the allele frequencies of 63,012 genic SNPs among 4 different populations (Hispanics, African Americans, Asian Americans and European Americans) show that, although most SNPs (from 72% to 96%) are present in the two compared populations, only 44% to 72% of these shared variants are found to have allelic frequencies >10% (i.e. to be common) in both populations [16]. Furthermore, a resequencing survey in a sample of 90 individuals from 6 world-wide populations showed that only 56% of common SNPs were already present in the HapMap database [17]. Finally, 25 out of 43 meta-analysis of complex disease-associated variants showed heterogeneity in allelic frequency among human populations [18].

It is thus reasonable to hypothesize that differences in the evolutionary history of loci associated to disease could have led to a non-homogeneous world-wide distribution of genetic risk variants. In this scenario, replication studies of risk alleles would frequently fail because of a true heterogeneity in the genetic architecture of common diseases. Previous studies have partially addressed the role of heterogeneity of the genetic ancestry in association studies, without positive results. Lohmueller et al. [19] analyzed population differentiation patterns between populations of European and African ancestry in 48 highly replicated disease-associated SNPs. Also, Myles et al. [20] analyzed the world-wide allelic distribution of 25 disease-associated SNPs from the WTCCC genome-wide scan [5]. Finally, Adeyemo et al. [21] checked for the differences in allele frequencies among 11 HapMap populations for 621 SNPs that had been associated to disease in GWAS performed with peoples from European ancestry. In all three studies, with the exception of some extreme differences in a few variants, disease-associated SNPs presented levels of differentiation among populations that were equivalent to the genome-wide average.

To date, however, no general study has tested whether inter-population genetic heterogeneity has affected the replication rates of association studies. Here, we aim to evaluate such a hypothesis. Ideally, the study should be carried-out by means of a comprehensive meta-analysis of GWAS data. However, there is still a bias in the populations that are chosen to perform these kind of association studies, since the great majority of them (≈90%) has been carried out upon individuals of European ancestry [22]. In addition, most of these GWAS use mixed panels of individuals from different regions in Europe, making it impossible to assign the status of replication of disease variants through populations within Europe.

In contrast, classical association studies based on candidate genes have been performed in great numbers all over the world and their results are publicly available. The Genetic Association Database (GAD) [23], is one of the largest repositories of the association studies carried out during the last 25 years. Analyzing that dataset, we find that risk variants from genes that diverged most between human populations present lower rates of replication. In contrast, world-wide distributed risk alleles appear to be located in loci that do not show population-specific patterns of genetic variability. These results point towards a role of the recent evolutionary history of human populations in shaping genetic risk for complex diseases and suggest that part of the disease variants that have not been replicated might be true risk alleles, at least in some populations.

## Results

**Figure 1:**
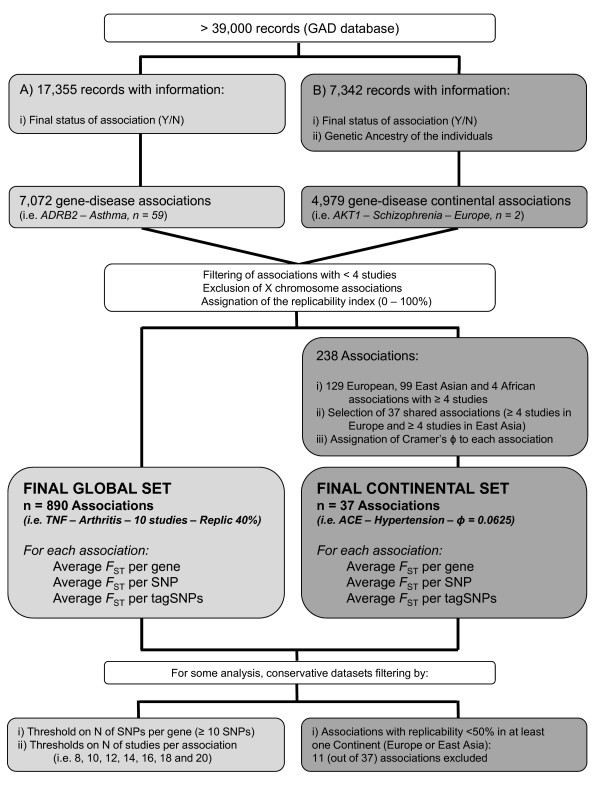
**Summary of the steps and filters to ascertain the Global and Continental Sets**. A further text summary is available in Additional File 1.

Two different sets of associations between genes and diseases were obtained from the Genetic Association Database [23]. The first set, named the Global Set, contained associations that had been replicated many times (at least 4 studies per association, n = 890), regardless of which human population had been tested in each study. The second set, the Continental Set, contained those associations that had been widely studied in both European and East Asian samples (at least 4 studies in each continent, n = 37). A summary of the main steps and filters to ascertain the Global and Continental Sets is available in Figure 1 and Additional File 1. Both Sets are listed in Additional File 2 and 3, along with their main features, such as the global replicability, continental-specific replicabilities and the degree of population differentiation corresponding to each association.

### Analyses of the Global Set - Global vs. Pairwise *F*_ST_

A first analysis showed that the disease-associated genes contained on the Global Set (n = 403 genes) present significantly lower inter-population genetic differentiation than equivalent sets of autosomic genes (*F*_ST _= 0.083 vs. *F*_ST _= 0.1045, resampling test, p-value < 10^-4^).

Next, we analyzed the relationship between levels of population differentiation and the replicability of disease associations. We detected a tendency towards negative correlations between *F*_ST _and replicability. The tendency is only visible when testing the most reliable associations, the ones with many studies or with longer genes (Additional Files 4, 5 and 6) and it maintains regardless of the method used to compute *F*_ST _(average genic *F*_ST_, by SNP or only tagSNPs). Thus, although there was a trend towards lower replicability of associations between disease and genes with high global *F*_ST_, most correlations were non-significant and the correlations between replicability and global *F*_ST _lacked consistency.

**Figure 2:**
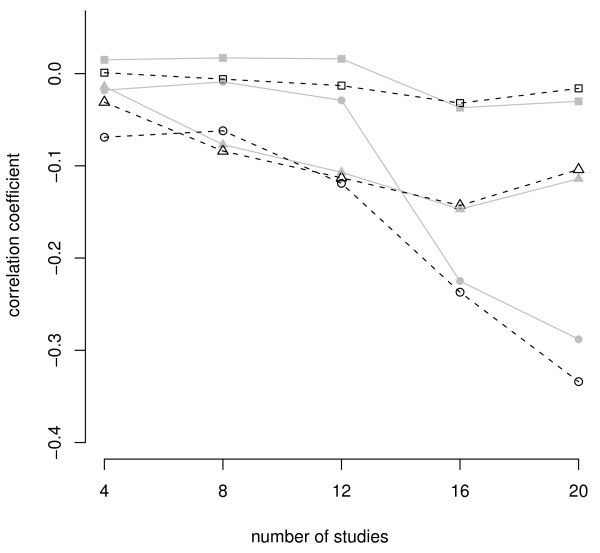
**Coefficients of correlation between replicability and *F*_ST _for the 890 associations from the Global Set**. Values correspond to Spearman correlation coefficients (ρ). The replicability is measured in % of positive studies over total studies and the genetic differentiation values correspond to average *F*_ST _values between Europeans and East Asians, under different pooling conditions. X-axis values indicate the cutoff of number of studies per associations, filtering out those associations with fewer studies than indicated. Solid grey lines correspond to the Full Set of associations. Dashed lines correspond to associations from the Conservative Set (replicability ≥50% in at least one continent). Circles, triangles and squares correspond to *F*_ST _values computed from average of the SNPs in a gene, individual SNPs and tagSNPs, respectively.

We performed a similar analysis focusing on pairwise *F*_ST _values. When values involved African individuals (European - African *F*_ST _and East Asian - African *F*_ST_) there was no clear pattern (Additional File 4 and 5). In contrast, our replicability measures consistently showed significant negative correlations with *F*_ST _values between European and East Asian populations (Additional File 4 and 5). Moreover, this pattern became more apparent after filtering out those associations that had been studied fewer times, that is, when using more reliable data (Figure 2). These results fit a well-known continental bias in the origin of samples: most of the associations reported in the GAD (≥94%) had been performed with individuals of European or East Asian ancestry. Thus, replicability indexes for the associations in the Global Set mostly reflect the outcome of studies upon these two continental populations, and it makes sense that they are related to pairwise *F*_ST _values between Europeans and East Asians and not to global *F*_ST _values, that include African individuals.

### Analyses of the Continental Set

**Figure 3:**
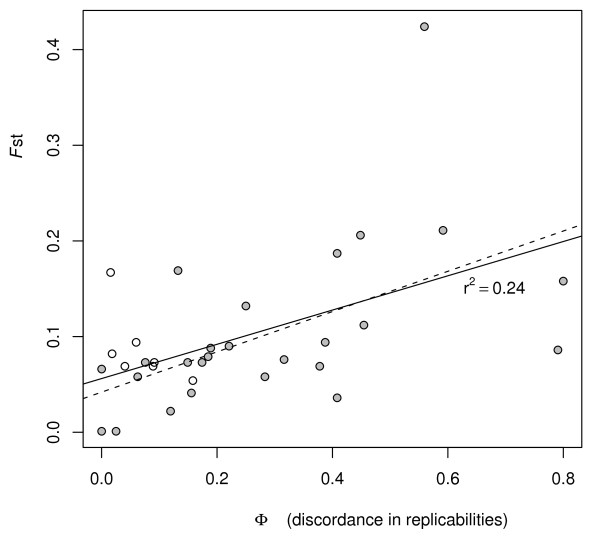
**Correlation between discordance in replicability and *F*_ST _for the 37 associations from the Continental Set**. The discordance in replicability values correspond to values and the genetic differentiation to average genic *F*_ST _values between Europeans and East Asians. Grey circles correspond to the associations from the Conservative Set (n = 26, replicability ≥50% in at least one continent). Solid line indicates the regression line for the full set of associations (n = 33). Dashed line indicates the regression line for the conservative set of associations.

Given the continental bias in the origin of studies, the Continental Set is more adequate to test our hypothesis since it includes only studies performed upon cohorts of European or East Asian ancestry. For the 37 associations in the Continental Set, the discordance in continental replicability measured by *ϕ *(see Methods) showed a consistent pattern of positive correlation with *F*_ST _values between Europeans and Asians (Table 1). In other words, genes with lower *F*_ST _formed associations with higher consistency of replicabilities between continents. This trend was detected under a diversity of approaches. First, a significant and positive correlation was detected when using average genic *F*_ST _values (ρ = 0.496, p < 0.003, n = 33, Figure 3). Second, the result maintained when performing a SNP-centered analysis in which each SNP was assigned the ϕ value corresponding to the gene in which it lay (ρ = 0.155, p < 10^-7^, n = 3,710). Moreover, because almost 80% of SNPs studied belonged to only two associations with very large genes (*NRG1 *- Schizophrenia/*PARK2 *- Parkinson's disease) a third analysis removing these two genes was performed and the same correlation between *F*_ST _and ϕ was detected (ρ = 0.152, p < 10^-5^, n = 821). In addition, *F*_ST _values obtained using only tagSNPs from the studied genes also showed a positive correlation to ϕ (ρ = 0.16, p < 2.2 × 10^-4^, n = 538), which maintained after removing the two largest genes. Finally, the positive correlation between *F*_ST _and discordance in continental replicabilities became even stronger when using the conservative strategy of keeping for analysis only the most reliable associations (with >50% replicability in both continents, Table 1).

**Table 1:** Summary of Spearman's correlation coefficients between *F*_ST _and ϕ as the discordance in replicabilities for the 37 associations from the Continental Set

ASSOCIATIONS	CATHEGORY	**POOLING**^**a**^	**VARIABLE**^**b**^	N		*P *value
Full Set	All SNPs	By Gene	Average *F*_ST_	33	0.496 *	*0.003*
			
			Variance *F*_ST_	32	0.636 *	*7 × 10^-5^*
		
		Independently	Average *F*_ST_	3710	0.155 *	*2.58 × 10^-21^*
	
	tagSNPs	By Gene	Average *F*_ST_	33	0.187	*0.313*
			
		Independently	Average *F*_ST_	538	0.16 *	*2.02 × 10^-4^*

Conservative Set^c^	All SNPs	By Gene	Average *F*_ST_	26	0.666 *	*0.0002*
			
			Variance *F*_ST_	24	0.666 *	*4.5 × 10^-6^*
		
		Independently	Average *F*_ST_	2454	0.15 *	*0.628*
	
	tagSNPs	By Gene	Average *F*_ST_	24	0.453	*0.280*
			
		Independently	Average *F*_ST_	486	0.063 *	*6.85 × 10^-4^*

^a ^Pooling by gene tests average genic *F*_ST_, while in pooling independently each SNP has been assigned the replicability from the association it belongs to

^b ^Variable indicates which parameter (either average or variance in *F*_ST_) has been tested versus ϕ

^c ^Conservative set contains those associations that have a replicability of ≥50% in at least one continent

These results suggest that differences in the continental replicabilities of disease associations (in Europe and East Asia) tend to occur in disease-associated genes that show an increased amount of genetic differentiation between human populations. Still, different confounding factors could be shaping this correlation. For instance, a recent study based on HapMap data has shown that the degree of differentiation in the frequency of SNPs in different human populations depend on the functional role of the SNPs [24]. Within genes, for instance, non-synonymous SNPs show the lowest amount of genetic differentiation among populations while SNPs located in 3'-UTR, 5'-UTR and intronic regions show an increased level of population differentiation. This trend was also observed in our data: intronic SNPs have a mean *F*_ST _of 0.117 (n = 3,590, 96.9% of the total) while exonic (synonymous and non-synonymous) SNPs have a mean *F*_ST _of 0.063 (n = 63, 1.7% of the total). Therefore, variable contributions of different SNP classes to high and low replicabilities may be driving the correlations between *F*_ST _and ϕ. Moreover, the fact that the GAD database pools association studies performed during a wide range of years and under many different conditions (such as sample size) constitutes another potential source of confounding factors.

To try to control for these potential sources of bias, we performed a multiple forward stepwise regression analysis to determine which variable or combination of variables best explained variance in ϕ. We introduced eight possible predictors in the model, the average genic *F*_ST _together with seven potential confounding factors: (1) total number of SNPs in the gene (related to gene length); (2) the percentage of intronic SNPs; (3) total number of studies in the association; (4) total number of studies performed in Europe; (5) total number of studies performed in East Asia; (6) the average sample size of studies; and (7) the average year of study publication for each association. In total, 564 association studies were surveyed (Additional File 7).

Our analysis unveiled a significant model (*F*_df: 1,31 _= 24.641, p < 5 × 10^-7^, adjusted R^2 ^= 0.596, Table 2) with two significant predictor variables: the total number of SNPs in a gene (Beta = 0.613, p < 7 × 10^-6^) and the genetic differentiation between populations as measured by *F*_ST _(Beta = 0.456, p < 0.00033). Values of tolerance were high, so we could confirm that these two predictors were independent (*i.e*. not correlated). Since, as we saw above, *NRG1 *and *PARK2 *genes stood out because of their large number of SNPs (1,213 and 1,676, respectively), we carried out another multiple forward stepwise regression analyses without these two genes (n = 31 associations). In this case, a significant model emerged (*F*_df: 1,29 _= 16.097, p < 0.00039, adjusted R^2 ^= 0.335) with *F*_ST _as the sole predictor variable (Beta = 0.597, p < 0.00039) explaining ϕ. Correlations become even stronger when using only the 26 associations from the more conservative set that includes associations with at least 50% replicability in each continent (see Methods and Table 2). These results highlight the role of *F*_ST _explaining the consistency of replicabilities in different continents

**Table 2:** Summary of multiple regression analysis for the Continental Set.

a) FULL SET	**F **_**df:1,31**_	P value	**R**^**2**^	Beta	*P *value	**Tol**^**c**^
a.1) Two predictors (n = 33)^a^						
Gene Length (number of SNPs)	24.641	*5 × 10^-7^*	0.596	0.613	*7 × 10^-6^*	>0.93
				
*F*_ST _(population differentiation)				0.456	*0.00033*	>0.99

a.2) One predictor (n = 31;NRG1/PARK2 out)						
*F*_ST _(population differentiation)	16.097	*0.00039*	0.335	0.597	*0.00039*	-

b) CONSERVATIVE SET ^b^	F _df:1,24_	P value	R^2^	Beta	*P *value	Tol^c^

b.1) Two predictors (n = 26)^a^						
*F*_ST _(population differentiation)	26.709	*9.9 × 10^-7^*	0.673	0.64	*1.1 × 10^-5^*	>0.99
				
Gene Length (number of SNPs)				0.502	*2.14 × 10^-4^*	>0.99

b.2) One predictor (n = 24;NRG1/PARK2 out)						
*F*_ST _(population differentiation)	18.023	*0.00031*	0.428	0.673	*0.000314*	-

^a ^6 Excluded non-significant variables: a) percentage of intronic SNPs in the gene from each association, b) total number of studies of each association, c) total number of studies performed in Europe, d) total number of studies performed in East Asia, e) average study sample size of each association, and f) average year of study performance in each association

^b ^Conservative set contains those associations that at least in one continent have a replicability index of 50%

^c ^Tol = Tolerance

It is still possible that this correlation could have arisen due to pure lack of statistical power. For instance, an association study in East Asians could have failed to replicate a previous association found in Europeans if, with similar sample size, the tested genes harbored markers with lower allele frequencies in the replica population. We calculated the percentage of SNPs per gene from the Continental Set that happened to be very rare in a given continent while being common in the other, that is, the percentage of SNPs that are common in just a continent (see Methods). This percentage of extreme-frequency SNPs was not correlated with ϕ (ρ = 0.138, p < 0.443, n = 33), but it was positively correlated with *F*_ST _(ρ = 0.469, p < 0.006, n = 33). Additionally, we performed an additional multiple forward stepwise regression with the addition of this statistic as another explanatory variable for. However, the same models as above arose (see Table 2), this statistic being discarded as an explanatory variable of the ϕ. Thus, we can exclude the possibility that *F*_ST _explains the differences in replicability between Europe and East Asia just as a by-product of lack of statistical power.

Finally, to further validate the correlation between ϕ and *F*_ST_, we performed a marker-based analysis in which we studied the associated variants themselves and not the genes that contain them. After manual scrutiny of the 444 papers that reported the 37 associations in the Continental Set, we established the genetic marker had been analyzed in each study, and ascertained that 54 different SNPs that where associated in these studies where available for *F*_ST _analysis (Additional File 8). Again, we found a positive correlation between the discordance in continental replicabilities measured by ϕ and the *F*_ST _from the selected markers (ρ = 0.286, p < 0.036, n = 54).

### Ancestral and derived alleles

The correlation between lack of replicability and larger genetic differentiation of human populations that we report here may reflect differences in the evolutionary history of genes affecting complex disease. Such differences may have arisen under different evolutionary scenarios since the ancestors of human populations left Africa. These scenarios range from neutral evolution governed by pure genetic drift to processes of population-specific adaptation to new environments. An excess of high-frequency derived alleles may be indicative of a shift in allele frequencies, pointing towards an active role of population-specific phenomena. Thus, we compared the amount of high-frequency derived alleles among genes from the Global Set, according to their replicability (Table 3). We defined high-replicability associations as those whose replicability was above the median (66.7%). In all populations, high replicability genes presented increased levels of high-frequency derived SNPs (derived allele with an allele frequency >50%). This trend was stronger in non-African populations. In Europeans, the derived allele was the major one for 20.7% of SNPs from the high-replicability associations (compared to only 19.2% in low-replicability associations, p < 4.89 × 10^-5^, chi-squared test). Also, high-replicability associations carried an excess of high-frequency derived alleles (22.6% in Chinese and 22.3% in Japanese) compared to low-replicability associations (21.2%, p < 3.19 × 10^-4 ^and 20.9%, p < 1.43 × 10^-4^, respectively) in East Asian populations. Finally, although less pronounced, this pattern held in Africans (16.8% compared to 15.9%, p < 0.0144, chi-squared test). Gene-specific values are available at Additional File 9.

**Table 3:** Population-specific test on the long-term evolutionary status for the SNPs from the 890 associations from the Global Set.

	**Europeans (CEU)**^**a**^		**East Asians (CHB)**^**a**^		**East Asians (JPT)**^**a**^		**Africans (YRI)**^**a**^	
Replicability	**ANC**^**b **^**(%)**	**DER**^**b **^**(%)**	**ANC**^**b **^**(%)**	**DER**^**b **^**(%)**	**ANC**^**b **^**(%)**	**DER**^**b **^**(%)**	**ANC**^**b **^**(%)**	**DER**^**b **^**(%)**
≤66.67%	19017	4533	18529	4991	18623	4919	19816	3757
(n = 441)	*(80.8)*	*(19.2)*	*(78.8)*	*(21.2)*	*(79.1)*	*(20.9)*	*(84.1)*	*(15.9)*
≥66.67%	19783	5173	19271	5620	19334	5556	20789	4187
(n = 441)	*(79.3)*	*(20.7)*	*(77.4)*	*(22.6)*	*(77.7)*	*(22.3)*	*(83.2)*	*(16.8)*
	p-value	*4.89 × 10^-5^*	p-value	*3.19 × 10^-4^*	p-value	*1.43 × 10^-4^*	p-value	*0.0144*

^a ^CEU = North Americans (Utah) of Northern European ancestry; CHB = Chinese from Beijing; JPT = Japanese from Tokyo; YRI = Yorubans from Ibadan (Nigeria)

^b ^ANC = SNPs with an ancestral major allele (freq ≥ 0.5); DER = SNPs with a derived major allele (freq ≥ 0.5)

## Discussion

We have analyzed the role of genetic heterogeneity among human populations in the replicability of genetic association studies. To address this question, we have measured the degree of population differentiation in loci that have shown differential patterns of association to disease, as reported in the Genetic Association Database [23]. We report three main results. First, SNPs harbored in genes associated with complex disease present lower *F_ST _*values than the rest of genic SNPs in the genome; second, there is a negative correlation between the replicability of studies associating genes to disease and the *F*_ST _values of the associated genes in European and East Asian populations; and, third, in the same populations, high replicability genes present increased levels of high-frequency derived alleles. These findings would confirm the importance of the recent evolutionary history of our species in the current patterns of susceptibility to complex diseases.

Given the large number of false positives reported in association studies [4,6-8] a relevant starting issue is the adequacy of the GAD to perform our analysis. In that respect, two points must be noted. First, it is important to see that replication studies, which are the center of our manuscript, are in fact a way to assess how likely previous associations are false positives. A good part of our study would be unnecessary if every association ever reported had been a true positive. In that sense, the known presence of both false and true positives in the database prompted the particular series of analysis that we presented here. The approach will be different when enough GWAS data are available, since, given current standards in the field; it is false negatives that dominate in these studies [6,25,26]. Secondly, even if the "low replicability" category contains a mixture of false and true positives, it is clear that the studies with highest replication rates will correspond to true positives. Indeed, it has been known for quite some time that a considerable number of genetic variants have been consistently associated to complex diseases. For example, a review of 25 associations by Lohmueller et al. [27] found an excess of replications in classical association studies that cannot be explained by false positives. Moreover, a recent paper by Siontis et al. [28] shows that a good number of the associations detected in non-GWAS classical association studies (mostly those extensively studied) have been replicated in recent GWAS (41 of 291 with a p < 10^-7^). Neither of these results would have been obtained if highly replicated associations would have been false positives.

Our first observation of lower *F_ST _*values in genes associated to complex disease is relevant to the adaptive history of these genes. It is well-known that purifying selection is the main force driving the evolution of genes related to Mendelian disorders, as they tend to harbor lower levels of polymorphism. In contrast, complex-disease associated genes seem to be under different pressures, with mixed evolutionary signals [29]. Overall, our observation of lower levels of population is suggestive of purifying selection. These findings contradict results from other authors that did not detect differences in *F*_ST _values of disease-associated variants relative to genome-wide levels [19-21]. However, these previous studies focused in variants instead of genes and, therefore, could only muster small sample sizes. Myles et al. [20] and Lohmueller et al. [19] studied, respectively, 25 and 48 SNPs, with the resulting lack in statistical power. More recently, the study by Adeyemo and Rotimi [21] was able to collect 621 disease-associated SNPs. As expected, they found both SNPs with very large and very low *F*_ST _values through populations. However, they focused on average *F*_ST _values per disease and did not test their global average *F*_ST _of 0.105.

Anyhow, our finding of low average *F*_ST _values in 403 genes that have been associated to disease is still inconclusive. Since our data mainly come from classical (non genome-wide) association studies, our observation may have different causes, some of them spurious. Of course, a true extensive role of purifying selection governing the evolution of these genes is a possibility; but it is also possible that certain classes of genes with particular average selective pressures tend to be involved in complex diseases; or that there has been a human bias towards the inclusion of certain categories of genes in association studies [30]. Indeed, when tested for functional enrichment of PANTHER Biological Process categories (see Additional File 10), complex-disease genes from the Global Set showed an enrichment for the category "Immunity and defense" (corrected p < 2.11 × 10^-40^) and an array of "signaling"- related categories, such as "Signal transduction", "Cell surface receptor mediated signal transduction" and "Cell communication" (corrected p-values = 9.71 × 10^-40^, 2.22 × 10^-30 ^and 1.21 × 10^-23^, respectively), but these results can be the consequence of anyone of the causes mentioned above, or of several of them.

In a previous analysis of the Genetic Association Database, Amato et al. [31] found a trend that seems opposite to the one we report here. Namely, they detected increased levels of population differentiation in disease-associated genes when compared to genome-wide base levels. However, a careful analysis shows that our results are consistent with Amato et al.'s and that the apparent contradiction is due to their analysis criteria differing from ours in two key aspects. First, their set of "disease genes" was composed by genes positively associated to disease at least once while, to avoid noise, we only included associations that had been studied four or more times (n = 1,793 vs. n = 403). Second, Amato et al. [31] used as the *F_ST _*value representative of each gene the maximum *F_ST _*value of any of the SNP within that gene. In contrast, we averaged the *F_ST _*values of all the SNPs in a gene. This second difference is crucial: when we repeat our analysis using the "maximum *F_ST_*" method we do find marginally significant increased levels of population differentiation in disease genes (*F*_ST _= 0.366, n = 403 vs. *F*_ST _of 0.345, n = 18,671, p-value < 0.022, Mann-Whitney test). The reverse is also true, when we analyze the gene set from Amato et al. [31] with our "average *F_ST_*" approach, we detect significantly lower population differentiation than genome-wide autosomic levels (*F*_ST _= 0.097, n = 1,631 vs. *F*_ST _of 0.104, n = 17,443, p-value < 4.4 × 10^-5^, Mann-Whitney test).

The fact that using either "maximum *F*_ST_" or "average *F*_ST_" leads to different results, raises the question of which approach is more accurate. We believe our method to be more precise, due to the larger average length of "disease genes". As such, they tend to harbor more SNPs than the average gene (34.8% more, with an average of 101.48 SNPs, n = 403 vs. an average of 75.28 SNPs, n = 18,671, p-value < 3.1 × 10^-14^, Mann-Whitney test). And, in fact, there is a strong positive correlation between the number of SNPs a gene harbors and the maximum *F*_ST _value these SNPs can reach (ρ = 0.527, p < 10^-50^, n = 19,074), while the correlation is much weaker with the gene-specific average *F*_ST _(ρ = 0.094, p < 10^-39^, n = 19,074). As a result, the maximum *F*_ST _is more biased by gene length than the average *F*_ST_. Therefore, an approach based on the average *F*_ST _in our data seems to be more accurate, in the sense that the average *F*_ST _of a gene is a better proxy of the amount of genetic differentiation at a given locus.

Our second main observation is that genetic heterogeneity through human populations varies greatly amongst loci associated to complex diseases. These loci present different degrees of population differentiation if we attend to their replicability and the consistency of replicabilities between Europeans and East Asians. These two populations are more similar for loci that contain variants which have been similarly associated to disease over and over again in different studies, while greater genetic differences are found in loci whose disease variants have not been consistently replicated. These observations can have at least three sources. First, it is possible that different statistical power in different populations is contributing to the correlation between continental replicability and *F*_ST_. For this to happen, it should be the case that genetic variants that have been associated to disease in a given population tend to be rare other parts of the world. However, we found no evidence of loci with low consistency of replicability having more SNPs with extreme frequencies (common in a population while rare in the other). Alternatively, recent theoretical studies demonstrate that rare variants may create spurious or synthetic associations at certain common alleles [32]. If rare causal variants make a substantial contribution to disease risk and if different populations present different genealogies, the spurious associations detected in each population would differ and replicability patterns may differ. This scenario would point to an important role for rare variants in the etiology of complex diseases. However it is difficult to see how highly replicated associations could be spurious and we did observe a stronger correlation between *F*_ST _and consistency of replicability for associations that have been replicated in at least 50% of the studies. The final explanation would be that certain variants are contributing to the risk for the disease in some populations but not in others. The range of factors underlying this possibility is not limited to purely genetic causes. For instance, some gene-environment interactions that have appreciable joint effects in complex diseases have been described [33] and environmental conditions vary widely across the planet. Thus, environmental variability among populations could have a role in the differential effect of genetic variants through populations that we have detected. In any case, the evolutionary history of humans would be such that some of the variants associated to disease would increase susceptibility differently in different populations.

Our study points at the heterogeneous genetic architecture of complex diseases, which even if modulated by similar cellular and molecular pathways in all humans, may present intricate population differences regarding causal variants and loci. Although in most cases the behavior of susceptibility or protective risk variants are shared through populations [18], some differential effects for the same alleles in different populations have been established, like the European-specific protective effects to HIV1 infection progression by the 32-bp deletion allele of the *CCR5 *gene [34-36] or the presence of two different haplotype blocks in the *NRG1 *gene that give susceptibility to schizophrenia in European and East Asian populations, respectively [37]. These differences could eventually lead to systematic differences among human populations in susceptibility to, and may underlie well-known cases, such as the differential susceptibility and prevalence of asthma between individuals of Mexican or Puerto Rican ancestry [38-40].

Usually, lack of replication of association mapping methods is thought to be due to the presence of confounding factors such as population stratification, lack of statistical power or publication bias. Therefore, stringent replication criteria are necessary to avoid false positives and to ultimately confirm that a certain genetic variant confers susceptibility to disease [4]. However, the fact that the allelic architecture of disease may be different through human populations raises the issue of revisiting some genetic association studies for complex diseases, since some putatively false positives might hint at diseases whose etiology is geographically heterogeneous.

As to the causes of these differences, it has been previously shown that there is variation in the disease-susceptibility variants that are present in different populations. These differences have been attributed to changes in selective pressures over standing variation [41,42] or to population-specific selective processes [43,44]. Our results showing that, when compared against low replicability genes, high replicability genes present lower *F*_ST _values between European and Asians, but high *F*_ST _values between either of these populations and Africans; together with the fact that derived alleles are more frequent in these high replicability genes in Asian and European populations, suggest that replicability has been higher in loci whose allele frequencies changed in the ancestors of Europeans and Asians after they left Africa. It is tempting to speculate about a role of natural selection in shaping this pattern, which would fit into suggestions about selection leading, in some cases, to disease as a side-effect consequence of adaptation [41,42]. However, our results could be just due to the action of genetic drift relaxing purifying selection in non-African populations. In fact, it has been shown that the bottleneck due to the out-of-Africa event induced a decreased ability of purifying selection to purge deleterious alleles [45].

## Conclusions

In summary, our results not only show that the evolutionary history of disease-associated loci (influenced either by demographic or by selective forces) plays a role in the genetic susceptibility to disease in Eurasians; but they also cast doubts about the status of false positives of many associations that have not been widely replicated. Obtaining this picture has only been possible by analyzing more than 20 years worth of classical association studies. We hope that the extension of GWAS to populations of non-European ancestry will allow, in time, to perform systematic research on the world-wide distribution of genetic risk variants.

## Methods

### Database

We used the Genetic Association Database (GAD, http://geneticassociationdb.nih.gov/, update December 29^th^, 2007) [23], comprising over 39,000 records, to select genetic loci that contain variants associated to common diseases. The GAD reports the most important features of genetic association studies published over the last 25 years, including, among others, risk variant, gene name, disease phenotype, sample ethnic origins, known epistatic interactions, conclusion of the study, journal, year and submitter. Every record refers to an association, that is, if a given study analyzes *k *different markers from the same gene, GAD keeps them into *k *different records performing *k *different associations. However, the protocols of the GAD are hierarchically gene-centered, with less than a 10% of the records providing systematic information about the actual marker analyzed. In other words, the database does not focus on studies of certain genetic markers but on associations between genes and disease phenotypes. Therefore, although ideally our aim was to distinguish marker-specific replicability patterns, we focused onto associations among genes and diseases. A summary of the steps and filtering undertaken upon the records from the GAD that are explained in following sections is available in Figure 1 and Additional File 1.

### First set of associations - Global Set

From the original database, we loaded in a local mySQL database those records (n = 17,355) that carried information on the final status of the association, with two possible states: positive or negative (association or lack of it, respectively). Then, all the associations between gene and disease (e.g., *CTLA4 *- diabetes type II) were selected. Next, we performed a global manually-controlled accuracy control to solve problems due to extra-sensitivity of our queries. Thus, those associations between the same gene and the same disease previously classified as different (such as "*NOS3 *- high blood pressure" and "*NOS3 *- hypertension") were clustered together. Also, typographical errors (e.g. "epilpsy" - "epilepsy") were corrected. At this point, our database was formed by 7,072 different associations between one gene and one disease. Although many associations had been studied several times (e.g., *ADRB2 *- Asthma, 59 times), most of them had been performed only once (4,491 associations, 63.5%).

### Second set of associations - Continental Set

From the original database, we kept those records (n = 7,342) for which besides the final status of the association (Y/N), there was also information on the ancestry of the samples (e.g., European Americans from New York). For instance, four different records tested for association between markers at the *AKT1 *gene and schizophrenia: three of them were positive and based on individuals from Iran, Japan and the USA, while the last study, performed with Finnish individuals, was negative (GAD ID: 116446, 116448, 144228 and 144230, respectively).

Next we classified each study according to the geographic origin of the individuals that took part in it. Incorporating consensus information on human evolutionary history [46,47], six major geographic regions were considered: Africa, Europe, Middle East, East Asia, Oceania and America. For example, the four studies from the association between *AKT1 *and schizophrenia were classified into three categories: those performed upon Finnish and USA individuals from European ancestry were grouped together and labeled as European (*AKT1 *- Schizophrenia - Europe - 2 times); the study with Japanese individuals was labeled as East Asian (*AKT1 *- Schizophrenia - East Asia - 1 study) and the study with Iranian individuals was classified as Middle Eastern (*AKT1 *- Schizophrenia - Middle East - 1 study). More recent world-wide migrations were also considered (e.g. association studies on African American individuals were labeled as African). Moreover, we recovered further information from those studies that had an ambiguous label on the genetic ancestry of the samples (such as "Australian" or "Canadian") and only those for which more specific and unequivocal information was available were kept (e.g. the label "Caucasian" was assigned to European category). Finally, those studies performed on a mixed panel of samples from different ethnical origins (e.g. "British individuals from Caucasian and Indian origins") were classified under the label of "Mixed", unless the study carried separate information on the association status (positive/negative) for each of the ethnicities present in the samples.

At this point, the 7,342 records from the Continental Set were classified into 4,979 different associations connecting one gene and one disease and classified into continental populations: 2,136 associations were labeled as European, 1,775 as East Asian, 287 as Mixed, 131 as African, 65 as Middle Eastern, 39 as Amerindian, 11 as Oceanian and 535 were left unassigned.

### Replicability Index Assignation

To measure the replicability of a given association, we calculated the proportion of positive studies compared to the total number of studies of the association. However, since a reliable replicability index can only be estimated if associations have been studied several times, we defined an arbitrary cutoff of four studies, so that only associations that had been studied at least four times were considered. After applying these criteria, the Global Set was finally formed by the 890 gene-phenotype associations that had been studied at least 4 times (out of 7,072 initial associations, Additional File 2).

For the Continental Set, 238 associations (out of 4,979) remained after applying the same criterion of at least 4 studies per association. Most of the remaining associations had been carried out with individuals from Europe (n = 129, 54.2%) and East Asia (n = 99, 41.6%). Only a few association studies had been performed with African (n = 4, 1.7%) or Mixed (n = 6, 2.5%) individuals. Since 3 out of the 4 African associations (*FCGR2A*, *NOS2 *and *TNF *loci) were studies about malaria, which is endemic of African populations, we decided to remove them from our analysis and focus on associations that had been widely studied in both Europe and East Asia (≥4 times in each). Thus, the final Continental Set was formed by the 37 overlapping associations consistently studied in each European and East Asian populations (Additional File 3).

### Discordance Index for Replicability in the Continental Set - Cramer's Phi (ϕ)

We used Cramer's ϕ coefficient to calculate an index of discordance among the continental-specific replicabilities, so we could make use of the geographic information in the Continental Set. This statistic ranges from 0 to 1 and constitutes an unbiased estimator of the strength of association between two qualitative variables from a contingency table [48]. For the Continental Set, these variables were "continent" (Europe or East Asia) and "positive and negative studies within continent". When ϕ = 0 there is no association between the two variables, indicating that the two levels of replicability in the two continents under study were consistent (*e.g*. a replicability of 70% in European and 70% in East Asian populations). On the other hand, ϕ = 1 indicates that there is a complete association between the degree of replicability and the continent of origin of the studied populations, that is, that replicabilities were discordant between continents (*e.g*. the replicability was 0% in European studies and 100% in East Asian studies).

### Genotypes

SNP polymorphism data from HapMap Project Phase 2 (release 22, April 2007) [15] were selected to study genetic variability between human populations. Only genic SNPs as defined by ENSEMBL (Build 35) were ascertained for further analyses (n = 1,439,152 SNPs, from 19,176 genes). We downloaded all genotypes for all unrelated samples from the four HapMap populations: 60 CEU individuals (samples of Northern-European ancestry from CEPH panel), 45 JPT individuals (from Tokyo, Japan), 45 CHB individuals (from Beijing, China) and 60 YRI individuals (Yorubans from Ibadan, Nigeria). Following previous works, JPT and CHB samples were clustered together due to their close genetic relationships (90 individuals, ASN from now on) [49]. We identified a total of 50,317 SNPs located in genes reported in the 890 associations from the Global Set; and a total of 6,092 SNPs within the 27 genes from the 37 associations in the Continental Set (no SNPs were found in 4 genes: *APOE*, *HLA-DQA1*, *HLA-DQB1 *and *LTC4S*). Finally, those SNPs that were monomorphic in both European and East Asian populations were removed from the Continental Set (final set, n = 3,710 SNPs).

### tagSNP selection

Adjacent SNPs tend to be inherited together (these SNPs being in Linkage Disequilibrium or LD). Therefore, any measure of genetic differentiation calculated for a given SNP may be correlated with the signal from nearby SNPs, if in LD. Since our aim is to check the patterns of replicability and genetic differentiation at different genetic loci, variable SNP densities and LD patterns through different genes might cause some bias in our estimates. To avoid this, we ascertained sets of representative SNPs (tagSNPs) for each block of LD in the genes under study. We used SYSNPs browser http://www.sysnps.org, [50] that uses the Tagger algorithm [51], to select the tagSNPs of our interest. We tagged for each population (CEU, ASN and YRI) using an r^2 ^threshold of 0.8 and minimum MAF of 0.1, considering only SNPs with a minimum genotyping call of 75% of the individuals. Finally, we selected those SNPs that appeared to be tagSNPs in all three populations, with a final set of 6,582 and 538 tagSNPs for the Global and Continental Sets, respectively.

### Population Differentiation (*F*_ST_) Calculation

We used Wright's *F*_ST _[52] to measure genetic differentiation among populations. This statistic ranges from 0 to 1 and quantifies the amount of differences in allelic frequencies among populations and has been classically used to measure genetic differentiation between populations. Allele frequencies and measures of *F*_ST _[53,54] for each SNP were calculated with Arlequin v3.11 [55] as implemented in SNPator [56], using the genotypes from the ASN, CEU and YRI populations for the Global Set and from the ASN and CEU populations for the Continental Set. Therefore, for each SNP we calculated three pairwise *F*_ST _values (European-Asian, European-African and Asian-African) and a global *F*_ST _value including the three HapMap populations. To test for genetic differentiation patterns in different genes, we computed *F*_ST _in three different ways (1) averaging out the *F*_ST _values of all SNPs in a gene; (2) using separately the *F*_ST _value corresponding to each SNP and (3) using for each gene only the *F*_ST _values corresponding to its tagSNPs. Finally, to study how association studies performed in different continents could have failed to replicate due to lack of statistical power, we calculated the percentage of SNPs for each gene from the Continental Set that happened to be rare (MAF < 0.1) in a given continent while common (MAF > 0.2) in the other continental population (see Table 2).

### A marker-based analysis of the Continental Set

One of the pitfalls of the GAD database is that the actual markers tested in each study have been rarely recorded. Therefore, we focused on genes and summarized the replicability of each association by genes. However, the tendency of classical association studies to test a set of few markers may have affected our replicability measures. Thus, we decided to perform an analysis based on the actual tested markers that would help to validate our findings. As surveying all the papers that have been selected from the GAD seemed unfeasible, we focused in the 564 records (from 444 papers) that belong to the 37 associations from the Continental Set. For each record (see Additional File 7), we selected those variants that had been tested in at least 10% of the studies from each association. In total, we gathered 72 different polymorphisms. Of those, 54 were SNP markers. For each, we gathered allele frequencies for Europeans and East Asians from either public databases (HapMap, ALFRED or dbSNP) or, if not available, from the paper with the highest sample size for each Continental population. Similar to the gene-based analysis of the Continental Set, for each SNP we calculated the *F*_ST _between Europeans and East Asians. Finally, we assigned to each marker the ϕ value from the association it belonged to. All the features from the selected markers are available in the Additional File 8.

### Ancestral vs. derived alleles

To study the role of long-term evolutionary pressures in disease-associated loci and the replication of association studies, we inferred the ancestral-derived status of each SNP using a phylogenetic parsimony criterion by means of orthologous alignments with chimpanzee (*Pan troglodytes*) and macaque (*Macaca mulatta*). Using the Ensembl v49 BlastZ-net alignments [57,58] we reported the ancestral or derived status for the major allele (allele frequency ≥0.5) for all SNPs in each HapMap population (Additional File 9).

### Gene Ontology analysis

We used the service "expression data analysis" from the PANTHER database tools website [59]. This utility permits to "uncover statistically significant relationships between input data and gene or protein functions" [60]. We tested the whole list of complex-disease related genes from the Global Set (n = 403) versus the NCBI full set of genes. By means of a binomial test, we obtained a Bonferroni-corrected p-value for under- or over-representation of each functional category for all Biological Processes.

### A conservative dataset

In some analyses (were indicated in the text) we applied some further filters in order to be even more conservative. First, we eliminated associations that had failed to replicate at least 50% of the time after many attempts on the basis that these associations lacked credibility (after filtering, 710 associations remained in the Global Set and 26 associations in the Continental Set). In addition, for the estimation of the average genic *F_ST_*, we filtered out any gene that had less than 10 SNPs in order to get more reliable measures of genetic distances. Finally, for the Global Set we applied varying thresholds on the number of studies, filtering out associations with less than 8, 10, 12, 14, 16, 18 or 20 studies, respectively.

### Statistical analyses

Statistical analyses were performed using SPSS version 15.0 (SPSS, Inc., Chicago, IL) and using scripts in R v2.10.1 [61]. To check whether average *F*_ST _from disease-associated genes from the Global Set was significantly different than genome-wide average *F*_ST_, we ran a resampling test, with 10,000 sets of genes randomly chosen from the whole genome. Each set of genes had the same number of genes (n = 403) than our Global Set. Thus, we checked how many times 403 random genes chosen from the whole genome had an average *F*_ST _value equal or greater than the average *F*_ST _from the Global Set.

## Competing interests

The authors declare that they have no competing interests.

## Authors' contributions

UMM analyzed and interpreted the data and wrote the manuscript. OL helped to conceive the project, analyzed and interpreted the data and performed part of the statistical analysis. FCas, FCal, RF, EB and JB helped in the interpretation of data and the revision of the manuscript. CMS contributed to part of the statistical analysis. FS and HD participated in the Ancestral vs. Derived analyses. AN conceived the project, interpreted the data and wrote the manuscript. All the authors have given final approval of the version to be published.

## Supplementary Material

Additional file 1 **Text summary of the steps and filters to ascertain the Global and Continental Sets**. Click here for file

Additional file 2 **Main Features and Population Differentiation values in the selected 890 associations from the Global Set**. Click here for file

Additional file 3 **Main Features and Population Differentiation values in the selected 37 associations from the Continental Set**. Click here for file

Additional file 4 **Summary of Spearman's (ρ) correlation coefficients between the world-wide replicability (in %) and the average genic population differentiation (*F*_ST_) for the 890 associations from the Global Set**. Click here for file

Additional file 5 **Summary of Spearman's (ρ) correlation coefficients between the world-wide replicability (in %) and the average population differentiation (*F*_ST_) for the 50,317 SNPs from the Global Set**. Each SNP has been assigned the replicability of the gene they locate. Click here for file

Additional file 6 **Summary of Spearman's (ρ) correlation coefficients between the world-wide replicability (in %) and the average *F*_ST _for tagSNPs from the 890 associations from the Global Set**. Each tagSNP has been assigned the replicability of the gene they locate Click here for file

Additional file 7 **Main Features from the 564 studies of the 37 associations from the Continental Set**. Click here for file

Additional file 8 **Main Features and Population Differentiation values in the 54 SNP markers from the selected 37 associations from the Continental Set**. Click here for file

Additional file 9 **Main Features and summary of population-specific ancestrality status for the 890 associations from the Global Set**. Click here for file

Additional file 10 **Gene enrichment analysis for the PANTHER database "Biological Process" categories for the 403 genes from the 890 associations of the Global Set**. Click here for file
