# Supplementary material for: Recent human evolution has shaped geographical differences in susceptibility to disease
Source: BMC Genomics. 2011 Jan 24;12:55. doi: 10.1186/1471-2164-12-55 (PMC3039608; doi:10.1186/1471-2164-12-55)
Supplement: Additional file 1 — Text summary of the steps and filters to ascertain the Global and Continental Sets. [file 1471-2164-12-55-S1.DOC]

**Summary of the filtering process**

We distinguished two kinds of records from the GAD database. First, those that only carried information on the final status of association (n = 17,355). These records served to ascertain the Global Set. Second, those records that carried information on the final status of association and the population studied (n = 7,342), that served to form the Continental Set.

In both cases, we grouped the records by genes, getting association between genes and diseases (i.e. A2M – Asthma). After, for both datasets we ascertained those that had at least 4 records. At this point, we had ascertained 890 and 238 different gene-disease associations for the Global Set and the Continental Set, respectively.

**Global Set**

We calculated a replicability index for each of the 890 associations of the Global Set. To study the correlation of the replicability with the genetic differentiation from each gene, we calculated 4 different measures of *F*_ST_: Global *F*_ST_, incorporating the allele frequencies of Africans, Europeans and East Asians from HapMap, and the three pairwise *F*_ST_ for each pair of populations (Additional File 2).

Then, we calculated the correlation between the replicability index of each association and the population differentiation in each gene. For these four different *F*_ST_ values, besides of calculating the correlation between replicability and average *F*_ST_ per gene, we also calculated these correlations (i) without pooling the SNPs per gene and (ii) selecting only tagSNPs. Additionally, in some cases we filtered out those associations that had less studies of whose genes had fewer than 10 SNPs (Additional Files 4, 5 and 6).

**Continental Set**

Most of the 238 associations from the Continental Set had been performed over European (n = 129) and East Asian (n = 99) populations. As our aim was to study the degree of similarity of the replicability in each population, we ascertained those 37 associations that had been studied 4 or more times in each population (final Continental Set). For each of the 37 associations from this dataset, we calculated the ϕ, which measures (from 0 to 1) the degree of similarity of the replicabilities in each Continental population. Also, for each association, we calculated the pairwise *F*_ST_ between Europeans and East Asians from HapMap (Additional File 3).

We calculated the correlation of ϕ and the *F*_ST_. Finally, to exclude a spurious correlation between these two variables, we calculated a multiple linear regression analysis to find the best predictors of the ϕ, we gathered data for each association on seven different possible explanatory variables to build : (1) total number of SNPs in the gene (related to gene length); (2) the percentage of intronic SNPs; (3) total number of studies in the association; (4) total number of studies performed in Europe; (5) total number of studies performed in East Asia; (6) the average sample size of studies; and (7) average year of study publication for each association. In total, 564 association studies were surveyed (Additional File 8).
